# Electroencephalography based delirium screening in acute supratentorial stroke

**DOI:** 10.1186/s12883-024-03942-3

**Published:** 2024-11-13

**Authors:** Gesine Hermann, Friederike Baumgarte, Julius Welzel, Peter Nydahl, Gregor Kuhlenbäumer, Nils Gerd Margraf

**Affiliations:** Department of Neurology, Christian-Albrechts-University, University Hospital Schleswig-Holstein, Arnold-Heller-Straße 3, 24105 Kiel, Germany

**Keywords:** Delirium, Diagnostics, Encephalopathy, Stroke

## Abstract

**Background:**

Up to 25% of patients suffering from an acute stroke are diagnosed with delirium during the hospital stay, with older age increasing the risk. Generalized slowing in the electroencephalogram (EEG) supports the diagnosis of delirium. We examined the potential of single-channel EEG (DeltaScan^®^) as an easy-to-use device on intensive care units for detecting delirium. Our aim was to investigate characteristics of bihemispheric EEG recordings and single-channel EEG in patients suffering from strokes with and without delirium and to analyze the diagnostic accuracy of EEG-based diagnoses.

**Methods:**

Within the first five days after stroke onset, patients received single-channel EEG DeltaScan^®^ and a routine 21-channel EEG. The DeltaScan^®^ analyzes right sided fronto-parietal EEG using a proprietary algorithm focusing on polymorphic delta activity (PDA). In routine EEG the power spectral density (PSD) in predefined frequency bands was analyzed based on 2-minute eyes-closed resting state segments. EEG-analyses were conducted in MNE (v1.3.1) in Python (3.10) and RStudio (v4.2.1).

**Results:**

In 9 of 53 patients (52–90 years) delirium was diagnosed according to DSM-V criteria. Sensitivity of DeltaScan^®^ was 44% (95% CI = 15.3–77.3%), while specificity was 71% (95% CI = 57–83%). We found patients with right hemispheric stroke having a higher probability to be false positive in DeltaScan^®^ (*p* = 0.01). The 21-channel EEG based power analysis revealed significant differences in frontal delta and theta power between patients with and without delirium (*p* < 0.05).

**Conclusions:**

When EEG is used in clinical practice to support a delirium diagnosis in stroke patients, bihemispheric recordings are likely preferable over unilateral recordings. Slowing in the delta- or theta-frequency spectrum over the site of stroke may lead to false-positive results in single channel EEG based delirium scoring.

**Supplementary Information:**

The online version contains supplementary material available at 10.1186/s12883-024-03942-3.

## Background

Delirium has been listed as one of six leading preventable medical complications in the elderly [1]. According to the DSM-V it is defined as an acute cerebral dysfunction that is characterized by impaired attention, awareness and cognition and typically fluctuates in severity. In patients suffering from an acute stroke, preliminary studies report incidences up to 25% [2–4]. In clinical practice, the reliable and objective detection of delirium is still a major challenge, partly due to the variability of symptoms [5–7], which applies in particular to neurologically ill patients [8, 9]. At the same time, stroke-related deficits such as aphasia, dysphagia, or neglect are considered risk factors of delirium [2, 10]. Shi et al. (2012) showed patients with delirium after acute stroke having a 4.7-fold increase in mortality; Han et al. (2010) also reported worsened functional outcome and prolonged hospitalization durations. Early detection resulting in early therapeutic intervention is therefore essential [11, 12] and an important key to improve individual clinical patient outcome. To date, the diagnosis of delirium has been based solely on clinical parameters, being defined in the DSM-V and ICD-10 criteria (13; Word Health Organization (WHO), 1993). Diagnosis might be supported by the finding of slowing in electroencephalography (EEG) towards theta (4–8 Hz) and delta (1–4 Hz) frequency bands, which is associated with encephalopathic states and, within these, delirium [13, 14]. Even more specific for acute encephalopathic states, such as delirium, is polymorphic delta activity (PDA) [15], being characterized by differing amplitude, frequency (1–4 Hz) and topographic distribution [16]. The detection of such electroencephalographic patterns offers the possibility of an objective diagnostic supplement. However, the interpretation can be confounded by the presence of comorbid conditions, such as strokes, which also induce EEG alterations. Acute and chronic ischemia show regionally reduced activity in alpha and beta frequency bands in the EEG [17–19].

We investigated electroencephalography based bedside tests as objective diagnostic tools for delirium in patients with acute supratentorial stroke. The test reliability of single-channel EEG is examined and interpreted in the context of Fast Fourier Transform (FFT)-based power spectra of routine EEG, whose delirium-typical changes are well known, but which is more laborious to register. Specifically, we investigate whether the distinction between delirium and non-delirium in patients with supratentorial ischemic strokes based on single-channel EEG is consistent with the clinical diagnosis. In addition, we test whether the side of the infarct influences the test reliability of the single-channel EEG, which is recorded over the right hemisphere by default.

## Materials and methods

This investigator-initiated diagnostic accuracy study was conducted at the Stroke Unit of the University Hospital of Schleswig Holstein (UKSH), Campus Kiel. The study protocol, according to the declaration of Helsinki, was approved by the local ethics committee (D 459/20). Reporting is based on criteria of the Standards for Reporting Diagnostic Accuracy (STARD) statement [20]. Data are available for reasonable request.

### Participants and data acquisition

We included patients with suspected supratentorial ischemic stroke in the area of the middle (MCA), anterior (ACA) or posterior cerebral artery (PCA) or with microangiopathic infarcts with subcortical but also supratentorial localization, from the age of 50 years.

Inclusion criteria were a maximum of five days between the onset of stroke symptoms and data collection. If stroke diagnosis was not confirmed during subsequent treatment, patients were excluded secondarily. Patients with severe speech or language barriers, such as global aphasia or anarthria or RASS [21] scores ≤ 3 were excluded, as clinical diagnosis of delirium based on DSM-V criteria is not possible at this degree of impairment of cognition or vigilance. Dementia (score < 25 on the Mini Mental State Examination [22]) or a score < 19 points on the Montreal Cognitive Assessment (MoCA) [23, 24]) or delirium due to substance withdrawal have also been excluded.

Age, sex, days spent in hospital, National Institutes of Health Stroke Scale (NIHS-Scale) [25] on admission, current RASS score [21], comorbidities and medical treatment were collected for all patients.

Patients gave written informed consent if possible. Otherwise, consent was obtained from an authorized representative or legal guardian. The single-channel EEG device was provided free of charge (Prolira, Utrecht, The Netherlands). Prolira had no influence on the study protocol and the data analysis. Data collection took place between 05/2021 and 04/2022.

### Delirium diagnosis

A trained member of the study team screened patients for delirium using DSM-V criteria [26]. Attending physicians then classified patients as negative / positive / uncertain for delirium according to the criteria. Disagreement or uncertainty led to exclusion. Time between diagnosis of delirium and further technical assessment was a maximum of 45 min to minimize the probability of delirium fluctuation.

### EEG recordings

Single-channel (DeltaScan^®^) and 21-channel EEG were applied one after the other in randomized order. Subjects were instructed to sit or lie in a relaxed position with eyes closed.

### Single-channel EEG

DeltaScan^®^ (v.2.0) uses one bipolar channel (Pz-Fp2, reference: Fpz). Based on manufacturer-defined preprocessing and analysis, the device detects polymorphic delta activity (PDA) after high-pass filtering (0.125 Hz), notch filtering (50/60 Hz), equipment noise filtering (24/64 Hz and their harmonics) and an artifact reduction algorithm [16, 27]. Detection and quantification of PDA is performed for the first artifact-cleaned 96 s, resulting in an ordinal scaled score between 1 (no PDA) and 5 (maximum PDA) [27]. As recommended by the manufacturer, scores of 1 or 2 are interpreted as ‘unlikely delirium’, scores of 3 to 5 as ‘probable delirium’. Recordings had a length of 2 to 5 min. They were excluded if subjects were not calm or opened their eyes.

### 21-channel EEG

EEG was acquired (Neurofax EEG-1200, Nihon Kohden) over 10 min with eyes closed as resting-state using 21 channels, initially sampled at 200 Hz, and referenced to A1 and A2. Electrodes were placed according to the 10–20 layout. An impedance level ≤ 10 kΩ was achieved. Two-minute epochs of low artifact signal were analyzed using MNE toolbox (v1.3.1) [28] in Python (3.10). EEG-data was high-pass filtered to 1 Hz and referenced to average. Bad channels were detected using the PyPREP package (v0.4.2) using the PrepPipeline function (“pyprep.NoisyChannels”) with default settings [29]. Bad channels were not included in further analyses. A maximum of 5 channels were excluded. This was followed by applying an independent component analysis (ICA) using the infomax algorithm [30]. Artifactual independent components (non-brain labelled) were identified by using the IClabel package [31], and the corresponding activity was removed by excluding the respective components from back projection. Power spectral density (PSD) using Welch’s method [32] from 1 to 40 Hz was calculated per channel. These spectra were further processed by the FOOOF (fitting oscillations and one over f) package [33] to extract power values in predefined frequency bands (alpha 8–12 Hz, beta 15–30 Hz, theta 4–8 Hz and delta 1–4 Hz).

### Statistical analysis

Statistical analyses were done using R (Version 4.2.1; R Core Team, 2022) and IBM^®^ SPSS^®^ Statistics Version 22 (SPSS Inc., Chicago, IL, USA). For demographic data Wilcoxon rank sum test or Fisher’s exact test were used when appropriate.

## Results

### Demographics

**Fig. 1:**
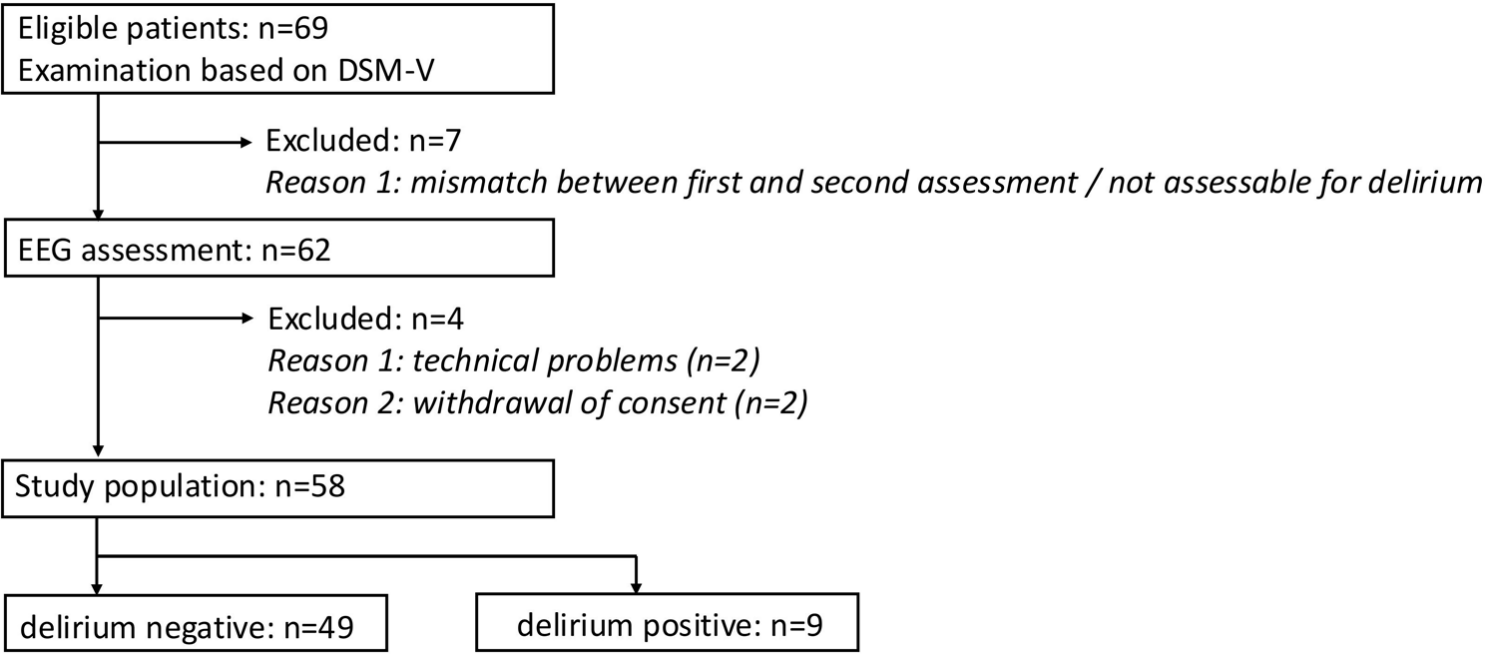
Exclusion criteria. Incongruence between the two DSM-V based evaluations for delirium were the most common reasons for secondary exclusion. From 69 patients enrolled in the study, a total cohort of 58 subjects was further analyzed

Out of 69 subjects, 11 (15,9%) were subsequently excluded, resulting in a total patient cohort of 58 (19 females, age mean 72 years, SD = 10.1 years). Out of 58 included subjects, 9 (15.5%) were assigned to the delirium group (3 hyperactive, 4 hypoactive, 2 mixed type). Figure 1 gives an overview of exclusion criteria.

Demographic data, comorbidity information, infarct patterns, and details of acute therapies are summarized in Tables 1 and 2. There were no significant differences in sex, age, NIH-SS, or comorbidities between patients with and without delirium (Table 1).

**Table 1:** Patient characteristics with mean ± SD, median and interquartile range or absolute and relative values as well as p-values and the used statistic tests: Fisher’s exact test for four-square tables; Mean, standard deviation and student t-test for normally distributed variables; median, interquartile range and Mann Whitney U test for non-normally distributed variables

	Non-delirium (*n* = 49)	Delirium (*n* = 9)	*p* values	test
Sex (female)	17 (34.7%)	2 (22.2%)	0.70	Fisher
Age (years)	71 ± 10	78 ± 10	0.09	t-test
Days spent in hospital	8 ± 7	11 ± 5	0.10	Mann-Whitney U
NIH-SS (points)	6 ± 6	7 ± 7	0.15	Mann-Whitney U
Arterial hypertension	28 (57.1%)	7 (77.8%)	0.76	Fisher
History of stroke	21 (42.9%)	4 (44.4%)	1.00	Fisher
Atrial fibrillation	17 (34.7%)	3 (33.3%)	1.00	Fisher
Diabetes mellitus I or II	11 (22.4%), out of these 1 type I	3 (33.3%), all type II	0.67	Fischer
Coronary heart disease	13 (26.5%)	2 (22.2%)	1.00	Fisher
Chronic kidney disease	4 (8.0%)	2 (22.2%)	0.23	Fisher
Chronic heart failure	4 (8.0%)	1 (11.1%)	1.00	Fisher
Depression	3 (6.1%)	1 (11.1%)	0.50	Fisher
Mild Cognitive Impairment	3 (6.1%)	1 (11.1%)	0.50	Fisher

**Table 2:** Distribution of stroke characteristics among 22 patients with right hemispheric strokes, 34 patients with left hemispheric strokes and 2 patients with bihemispheric strokes. The table shows the number of infarcts in the anterior (ACA), middle (MCA) and posterior cerebral artery (PCA) regions, as well as the number of microangiopathic subcortical infarcts. It further contains data on how many patients received thrombolysis or thrombectomy and their outcomes (TICI scores)

	Vascular territory	Thrombolysis	Thrombectomy	
Right hemispheric stroke *n* = 22	ACA *n* = 3	*n* = 0	*n* = 0	
	MCA *n* = 17	*n* = 5	*n* = 8	TICI 2a *n* = 1 TICI 2b *n* = 1 TICI 2c *n* = 2 TICI 3 *n* = 4
	PCA *n* = 1	*n* = 0	*n* = 0	
	microangiopathic *n* = 1	*n* = 0	*n* = 0	
Left hemispheric stroke *n* = 34	ACA *n* = 2	*n* = 1	*n* = 1	TICI 2b *n* = 1
	MCA *n* = 17	*n* = 4	*n* = 4	failed *n* = 1 TICI 2c *n* = 2 TICI 3 *n* = 1
	PCA *n* = 7	*n* = 2	*n* = 0	
	microangiopathic *n* = 8	*n* = 2	*n* = 0	
Bihemispheric stroke *n* = 2	MCA *n* = 1	*n* = 0	*n* = 1	TICI 3 *n* = 1
	PCA *n* = 1	*n* = 0	*n* = 0	

### Single-channel EEG

Distribution of DeltaScan^®^ scores ranging from 1 to 5 is presented in Table 3. Among 18 patients with increased scores (ranging from 3 to 5) and classified as “probable delirium”, 4 were clinically diagnosed with delirium, representing true positives in the contingency table (Table 4). In the control group (stroke without delirium, *n* = 49), 14 individuals received a positive DeltaScan^®^ result, giving a false-positive rate of 28.6%. Sensitivity of DeltaScan^®^ is computed as 44% (95% CI = 15.3–77.3%), while specificity is 71% (95% CI = 57–83%).

**Table 3:** Distribution of single channel EEG (DeltaScan^®^) scores for controls and delirium. DeltaScan^®^ gives nominal scores reaching from 1 (low) to 5 (high), which are indicated in the left column. Scores from 3 to 5 are interpreted as increased PDA and probable delirium

DeltaScan^®^ Score	Non-delirium (*n* = 49)	Delirium (*n* = 9)	Total (*n* = 58)
1	32 (55.2%)	3 (5.1%)	35 (60.3%)
2	3 (5.1%)	2 (3.4%)	5 (8.6%)
3	8 (13.8%)	3 (5.1%)	11 (19.0%)
4	3 (5.1%)	0 (0%)	3 (5.1%)
5	3 (5.1%)	1 (1.8%)	4 (6.9%)

**Table 4:** Contingency table for DeltaScan^®^ results (unlikely delirium: score ≤ 2, probable delirium: score > 2)

		Non-Delirium (Total, %)	Delirium (Total, %)	Sum of groups (Total, %)
DeltaScan^®^ result	Delirium negative	35 (71.4%)	5 (55.56%)	40 (69.0%)
	Delirium positive	14 (28.6%)	4 (44.44%)	18 (31.0%)
	Total	49 (100%)	9 (100%)	58 (100%)

### Interplay of infarct localization and single channel EEG

DeltaScan^®^ produced false positive results in 14 patients (28.6%). Analyzing these 14 subjects according to the hemisphere affected by infarction, 10 out of 14 (71.4%) suffered from right hemispheric strokes (MCA *n* = 9, ACA *n* = 1). Of the 35 subjects classified as true-negatives, 25 suffered from left hemispheric strokes. False-positive rate (FPR) differed significantly between groups of left hemispheric (FPR = 13,8%) and right hemispheric strokes (FPR = 52,6%) (*p* = 0.01).

### Parameters of the 21-channel EEG to distinguish between delirium and non-delirium

**Fig. 2:**
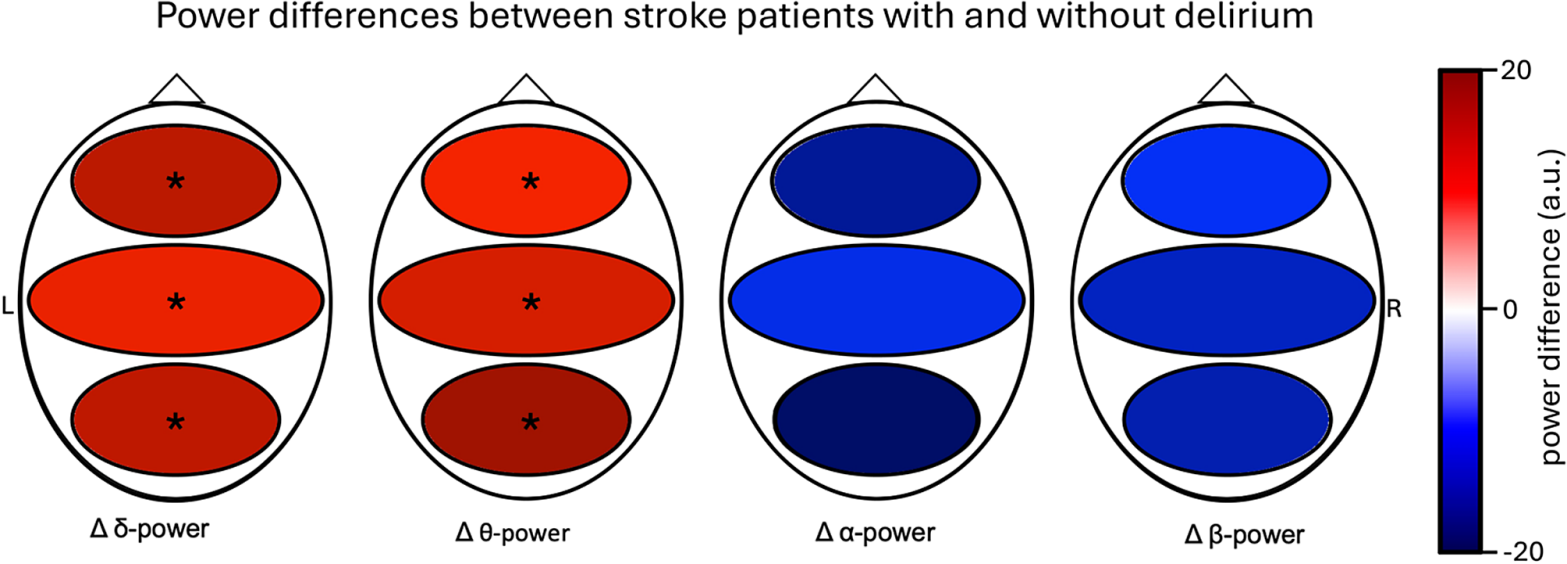
Patients with delirium show higher delta- and theta-power in all predefined regions (frontal, central, occipital). Frequency band specific (delta, theta, alpha, beta-power) power spectral density was calculated for each electrode. Mean value of power per region in the control group was subtracted from the mean value per region in the delirium group, giving the power difference (a.u.) between groups. Positive values (red) indicate higher power in the delirium group, whereas negative values (blue) indicate higher power in the control group. Asterisks indicate significant differences in FOOOF power between the two groups

We investigated whether and where frequency band-specific power of the 21-channel EEG differs between stroke patients with delirium and stroke patients without delirium. Significant differences in delta and theta power were found in all predefined cortical regions, both when examined individually for left and right hemispheres and when examined across both hemispheres (Fig. 2, Supplementary Table 1). While mean power values for alpha- and beta-frequency band specific power were higher in the control group, no significant differences were attained (Supplementary Fig. 1).

## Discussion

Based on data from a right fronto-parietal single-channel EEG (DeltaScan^®^) and a 21-channel EEG, we investigated discriminatory power for detecting delirium in 58 patients with acute stroke. We found that:

1. Single-channel EEG-based PDA analysis is probably ineffective as a screening tool for delirium in a stroke patient population.
2. The single-channel EEG-based PDA score exhibited a false-positive rate of 28.6%. This rate depended significantly on stroke localization (left versus right hemisphere, *p* = 0.01) and was higher for right hemispheric strokes.
3. 21-channel EEG based frontal power in theta- and delta-frequency bands significantly differed between patients with delirium and controls and was higher in the delirium group.

To our knowledge, this investigation is the first to explore discriminatory power of DeltaScan^®^ specifically in stroke patients. The reported test results question the usefulness of DeltaScan^®^ for clinical purposes in stroke patients. Nevertheless, performance must be viewed with caution given the small number of delirious patients (*n* = 9). The limited sample size may, in part, be attributed to the exclusion of patients with severe speech or language disorders, language barriers, or a RASS [21] score ≤ 3. Given the well-established association between aphasia or anarthria and increased risk of delirium [2, 8–10], as well as the potential exclusion of patients with severe hypoactive delirium, this may have impacted the DeltaScan^®^’s performance. It is conceivable that the exclusion of severely affected patients led to a reduction in performance because they could have been easily identified by the screening procedure. Nevertheless, this procedure was necessary to ensure a clear allocation to the delirium and non-delirium group.

As a result, precise evaluation of diagnostic properties of the DeltaScan^®^ for stroke patients remains elusive; however, in view of the upper confidence interval limits (sensitivity = 77.3%, specificity = 83%), test accuracy is probably not sufficient for clinical use in patients suffering from stroke. In contrast, the manufacturers describe a high sensitivity and specificity for the detection of EEG-based encephalopathy (sensitivity = 0.88, specificity = 0.88) and for delirium (sensitivity = 0.74, specificity = 0.73) [27]. Considering other existing studies, a low discriminatory power remains plausible [26, 34, 35]. Research that evaluated the diagnostic potential in intensive care patients without stroke or other acute cerebral lesions have shown low diagnostic performance of DeltaScan^®^ too. Aben et al. (2022) focused on EEG-based diagnostic accuracy for delirium in ventilated ICU-patients (*n* = 20, 75% delirium positive). No significant correlation between DeltaScan^®^ scores and DSM-V based delirium diagnosis was observed [26, 36].

Despite the limited sample size and the divergent test accuracies compared to the manufacturer’s studies, our findings also seem conceivable due to the significantly increased false positive rate for right hemispheric strokes [36, 37]. Focal EEG changes over the infarct site may contribute to misinterpretation of the DeltaScan^®^. We suspect that left hemispheric recordings would also show an increased FPR for left hemispheric strokes, which would likely reflect stroke-induced EEG slowing rather than underlying delirium. However, since the device is not approved for left hemispheric use, such data is unavailable.

Thus, despite the limited sample size and possible bias due to focal slowing, our findings remain concordant with existing literature. Most other groups investigated diagnostic value of single-channel EEG using raw signal data [19, 38, 39] rather than reducing it to a nominal scaled score, making direct comparison of test accuracy with these studies difficult. Right frontal delta power was found to differ significantly between groups without delirium, with possible delirium and delirium [19]. This is consistent with our 21-channel EEG data, which showed significant differences in frontal delta and theta power between groups, not only over the right hemisphere, but even independent of the side studied. Since DeltaScan^®^’s PDA-based algorithm is not publicly available, one can speculate whether a higher weighting of the absolute frontal delta power might provide an additional diagnostic benefit to the already considered variability of amplitude and frequency within the delta frequency band.

A direct comparison of test accuracy between a single-channel device and a 21-channel device is inherently challenging, mainly due to differences in data complexity, which results in different preprocessing (e.g. more channels = better artefact rejection). While more dimensional data can provide a more detailed picture of the brain’s electrical activity, it also increases the likelihood of overfitting, especially if the recording duration is not long or data is highly variable. Data multidimensionality aside, the manufacturer-based algorithm of DeltaScan^®^ was developed using an inhomogeneous cohort. In contrast, the cut-off values for delta power derived from 21-channel EEG data were established using only stroke patients, which may also lead to overfitting. Considering differences in false-positive rates and diminished performance in ICU patients, we emphasize that it is imperative to include the hemispheric side of the stroke and comorbidities known to cause EEG changes in the interpretation of results.

## Conclusions

DeltaScan^®^ demonstrated low diagnostic performance in our study, with a false positive rate of 28.6% and false negative rate of 55.56%. This was significantly influenced by the location of the stroke. In contrast, 21-channel EEG-based frontal power in theta and delta frequency bands was significantly higher in patients with delirium. Our findings are in line with the well-studied delirium-associated changes in routine EEG [40–43]. Based on our study, bihemispheric EEG recordings may be better suited than DeltaScan^®^ to support the diagnosis of delirium in stroke patients.

## Electronic supplementary material

Below is the link to the electronic supplementary material.

Supplementary Material 1: Figure 1. Percentage of frequency-band-specific power (delta, theta, alpha, beta frequency band) relative to total power across predefined regions (frontal, central, posterior) for both groups (red = delirium yes, blue = delirium no). Boxplots illustrate the median, interquartile range, and outliers, which are determined based on a function of the interquartile range

## Data Availability

The datasets generated and/or analyzed as part of the current study are not publicly accessible for the protection of patients but are available on reasonable request from the corresponding author.

## Abbreviations

ACA: Anterior Cerebral Artery
AUC: Area-Under-the-Curve
EEG: Electroencephalography
FFT: Fast Fourier Transform
FOOOF: Fitting Oscillations and One-Over-F
ICA: Independent Component Analysis
MCA: Middle Cerebral Artery
MoCA: Montreal Cognitive Assessment
NIHSS: National Institutes of Health Stroke Scale
PCA: Posterior Cerebral Artery
PDA: Polymorphic Delta Activity
PSD: Power Spectral Density
RASS: Richmond Agitation Sedation Scale
ROC: Receiver-Operating-Characteristic
STARD: Standards for Reporting of Diagnostic Accuracy Studies
UKSH: University Hospital Schleswig-Holstein
WHO: World Health Organization
