# Supplementary figures and images for: Electroencephalography based delirium screening in acute supratentorial stroke
Source: BMC Neurol. 2024 Nov 13;24:442. doi: 10.1186/s12883-024-03942-3 (PMC11558914; doi:10.1186/s12883-024-03942-3)

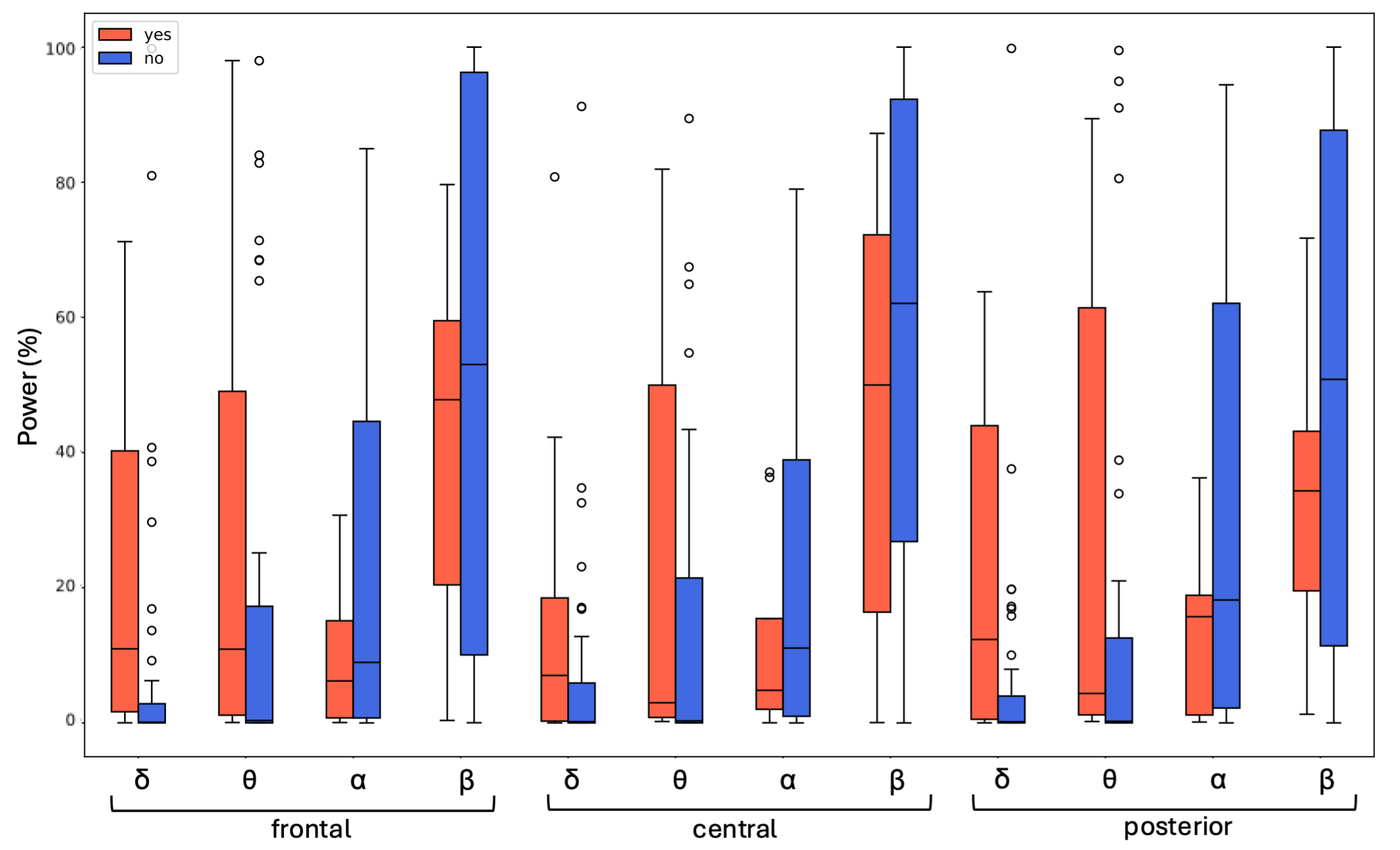

Supplement: Supplementary file 1 — Supplementary Material 1: Figure 1. Percentage of frequency-band-specific power (delta, theta, alpha, beta frequency band) relative to total power across predefined regions (frontal, central, posterior) for both groups (red = delirium yes, blue = delirium no). Boxplots illustrate the median, interquartile range, and outliers, which are determined based on a function of the interquartile range [file 12883_2024_3942_MOESM1_ESM.png]
